# Single-cell transcriptomic analysis of peripheral blood mononuclear cells reveals key immune responses in ST-segment elevation myocardial infarction

**DOI:** 10.1186/s41065-025-00609-y

**Published:** 2025-11-25

**Authors:** Zheng Zhang, Shengfang Wang, Yahui Liu, Gaohan Li, Qianqian Cheng, Wei Yang, Ganxin Yan, Chuanyu Gao

**Affiliations:** 1 Department of Cardiology, Central China Fuwai Hospital of Zhengzhou University, Fuwai Central China Cardiovascular Hospital, No. 1 Fuwai Road, Zhengzhou, Henan 451464 China; 2 Henan Provincial Key Lab for Prevention and Control of Coronary Heart Disease, Central China Fuwai Hospital of Zhengzhou University, No. 1 Fuwai Road, Zhengzhou, Henan 451464 China; 3 Lankenau Institute for Medical Research, Jefferson Medical College of Thomas Jefferson University, 100 Lancaster Ave, Wynnewood, PA 19096 USA

**Keywords:** ST-segment elevation myocardial infarction, Single-cell RNA sequencing, Peripheral blood mononuclear cells, Inflammation

## Abstract

**Background:**

Inflammation plays a crucial role in the pathogenesis of ST-segment elevation myocardial infarction (STEMI). However, the precise immunological mechanisms remain incompletely understood. Single-cell RNA sequencing (scRNA-seq) provides a powerful approach to dissect immune cell heterogeneity and dynamic changes at single-cell resolution.

**Methods:**

Peripheral blood mononuclear cells (PBMCs) were collected from 7 STEMI patients (within 6h after primary percutaneous coronary intervention) and 3 healthy controls. Single-cell suspensions were prepared and subjected to scRNA-seq using the 10x Genomics Chromium platform and Illumina NovaSeq 6000. Data were processed using Cell Ranger and analyzed with Seurat for quality control, clustering, and annotation. Differentially expressed genes (DEGs) were identified and analyzed through Gene Ontology (GO), Kyoto Encyclopedia of Genes and Genomes (KEGG), gene set variation analysis (GSVA), and gene set enrichment analysis (GSEA). Cell-cell communication was inferred using CellChat. And protein–protein interaction (PPI) network analysis was performed via STRING and Cytoscape. Key genes were validated using quantitative real-time PCR (qRT-PCR).

**Results:**

A total of 71,288 PBMCs were analyzed. Significant differences in immune cell composition were observed between groups, especially in monocytes and T cells. Monocytes were divided into 10 subclusters; subclusters 0, 1, and 6 showed marked expansion and were functionally associated with inflammation, antigen presentation, and cytokine regulation. PPI network analysis identified JUN as a key hub gene, which was confirmed by qRT-PCR. T cells were divided into 7 subtypes, and GSEA revealed enrichment of IL-2/STAT5 and TNF-α/NF-κB signaling in STEMI. CD69 and ICOS were significantly upregulated in T cells. CellChat analysis revealed enhanced intercellular communication in STEMI, with the CXCL signaling pathway (notably PF4–CXCR3 interaction) being highly upregulated.

**Conclusions:**

This study reveals key inflammatory and immune characteristics of PBMCs in STEMI patients. JUN and the CXCL signaling axis represent potential targets for immunomodulatory therapy in acute myocardial infarction.

**Graphical abstract:**

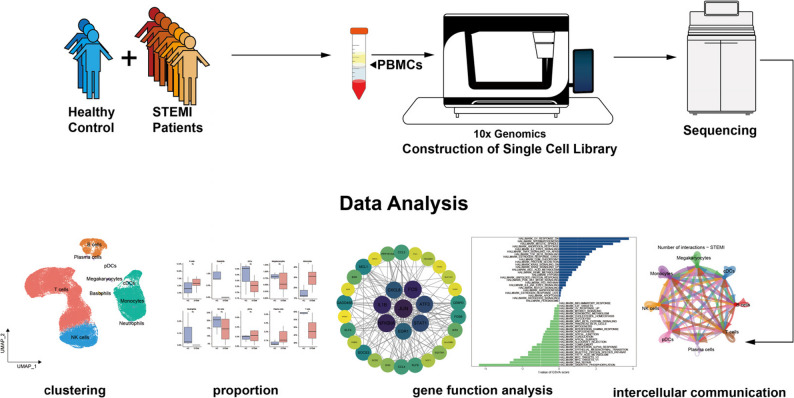

**Supplementary Information:**

The online version contains supplementary material available at 10.1186/s41065-025-00609-y.

## Introduction

ST-segment elevation myocardial infarction (STEMI) is primarily caused by the rupture of atherosclerotic plaques in coronary arteries, leading to complete vascular occlusion and rapid loss of myocardial perfusion. Despite advancements in STEMI treatment, including widespread application of primary percutaneous coronary intervention (PPCI) and modern thrombolytic therapies, the 12-month mortality rate remains as high as 8% [1, 2]. Inflammation plays a pivotal role in the development of coronary atherosclerosis, acute myocardial infarction (AMI), and subsequent ischemia-reperfusion injury (IRI) [3–5]. The immune system is deeply involved in the initiation and progression of atherosclerotic plaque formation, plaque rupture, and the resulting inflammatory cascade [6, 7]. Several clinical trials have explored anti-inflammatory strategies in STEMI, such as VCU-ART3, which investigated the effect of IL-1 blockade on acute inflammation [8], and ASSAIL-MI, which evaluated IL-6 inhibition [9]. However, due to the complexity of immune responses and potential adverse effects, targeting inflammation in STEMI remains a major challenge. A more in-depth understanding of the underlying immunopathological mechanisms is essential.

Peripheral blood mononuclear cells (PBMCs), comprising monocytes, T lymphocytes, B lymphocytes, natural killer (NK) cells, and others, are key players in the immune response. Previous studies have highlighted the role of PBMCs in the pathogenesis of AMI [10–12], making them a suitable surrogate tissue for investigating immune alterations in STEMI.

Traditional transcriptomic analyses relied on bulk RNA sequencing, which averages gene expression across heterogeneous cell populations. In contrast, single-cell RNA sequencing (scRNA-seq) enables high-resolution transcriptomic profiling at the individual cell level, allowing for the identification of distinct cell subtypes and states within complex tissues [13, 14].

In this study, we performed scRNA-seq of PBMCs from STEMI patients and healthy controls to comprehensively characterize immune cell heterogeneity, identify key gene expression changes, and uncover potential alterations in intercellular signaling pathways. These findings aim to provide novel insights into the inflammatory and immune responses following STEMI.

## Materials and methods

### Study subjects

STEMI patients were hospitalized in Fuwai Huazhong Cardiovascular Disease Hospital, which ranged from December 26, 2022, to May 25, 2023. The diagnosis of STEMI was made on the basis of raised cardiac markers (troponin T/I or creatine kinase-MB) and characteristic chest pain, as well as new ST-segment elevation of at least two contiguous leads > 0.1 mV on the 12-lead ECG. Severe infectious disease, autoimmune disease, malignant tumor, or sepsis were the exclusion criteria. The patients with STEMI had undergone PPCI and individuals in the HC group did not have coronary heart disease.

All experimental protocols were conducted in accordance with the Declaration of Helsinki and approved by the ethics committee of Fuwai Central China Cardiovascular Hospital (2022-Y003-02). Informed consent was obtained from all subjects of this study. Additional file 1 contained a summary of the fundamental data as well as the clinical examination.

### Isolation of PBMCs

All subjects had 4 ml of venous blood extracted; in the case of STEMI patients, the blood was taken no later than 6 h following PPCI. PBMCs were extracted from diluted layered whole blood by density gradient centrifugation at 800×g for 20 min at 4℃, after which PBMCs were separated from the middle white monolayer using Lymphocyte Separation Medium (STEMCELL, Canada). After gathering the mononuclear cells from the middle layer, the cells were cleaned, resuspended in PBS, and used for droplet of scRNA-seq.

### Preparation of single cell suspensions

Human PBMCs had been single-cell suspended using the 10× Genomics single cell procedures. The Countstar Rigel S2 automatic fluorescent cell analyzer was utilized to determine the viability and concentration of PBMCs. For fixation and scRNA-Seq analysis, PBMCs with a concentration of roughly 700–1200/µl and a viability of over 85% were stored on ice.

#### Single cell library construction and sequencing

Following the manufacturer’s instructions based on 10X Genomics’ exclusive technology, cellular suspensions were put into a 10X Chromium Connect (10X Genomics). Using the Chromium Single Cell 3′ v3 Reagent Kit (10X Genomics) and the manufacturer’s instructions, all scRNA-seq libraries were created. In summary, automated generation of gel bead-in-emulsions (GEMs) were done by combining barcoded single cell 3ʹ v3.1 gel beads, a master mix containing single-cell suspension and enzymes, and partitioning oil onto chromium next GEM automated Chip G. Poly(dT) sequences, unique primers with 10× cell barcodes, and unique molecular identifier (UMI) were coated on the gel beads. Immediately following GEMs generation, the gel beads were dissolved, primers released, and any co-partitioned cell was lysed. Primers were mixed with the cell lysate and a master mix containing reverse transcription (RT) reagents. Incubation of the GEMs produced barcoded, full-length cDNA from poly-adenylated mRNA. After incubation, GEMs were broken and pooled fractions were recovered. Silane magnetic beads were used to purify the first-strand cDNA from the post GEM-RT reaction mixture, which included biochemical leftover reagents and primers. Barcoded, full-length cDNA was amplified via PCR to generate sufficient mass for library construction.

After being fragmented, the amplified cDNAs were processed into final libraries appropriate for Illumina paired-end sequencing, where adaptor and sample index were added. The Agilent Bioanalyzer 2100 was used to analyze the size profiles of the final libraries and pre-amplified cDNA. The indexed libraries were pooled and sequenced on an Illumina NovaSeq 6000 using 150 bp paired end reads.

### Data preprocessing, alignment, mRNA quantification and quality control for scRNA-Seq

The sequencing data was analyzed with the Cell Ranger Pipeline (version 7.0.0), which was used for barcode processing, alignment and quality control. Spliced transcripts alignment to a reference (STAR) software was used to match the read 2 to the GRCh38-2020-A human reference transcriptome. The first steps in quality control and cell filtering were then completed. UMI and gene counts of each cell were measured, and this information was used to generate expression matrix files for additional analysis.

### Clustering and annotation of PBMCs

Seurat (version 4.3.0.1) was then used to import the gene-cell-barcode matrix files in order to do subsequent quality control and further analysis of the scRNA-seq [15]. First, the CreateSeuratObject function of the Seurat software package was used and the mitochondrial gene expression was determined using the PercentFeatureSet function [18]. The scDblFinder [16] R package (version 1.12.0) was used to filter the potential doublets. Cells were discarded according to the following criteria: (1) cells that had fewer than 1000 genes or more than 5000 genes; (2) cells that had fewer than 1000 UMI or over 20,000 UMI; (3) cells that had more than 10% mitochondrial UMI counts. Then, the function of NormalizeData was employed to normalize the feature expression measurements for each cell by the total expression and log-transform the result to yield the normalized UMI value. The function of FindVariableFeatures and ScaleData were used to further standardize the data. The harmony R package (version 1.2.0) was employed to remove the batch effect and integrate the single cell data. Then, the function of RunUMAP was used to visualize the uniform manifold approximation and projection (UMAP) using the top 20 dimensions from principal component analysis (PCA). After, the functions of FindNeighbors and FindClusters were further performed to identify clusters of cells by a shared nearest neighbor (SNN) modularity optimization based clustering algorithm. The tree plot for cluster analysis was performed by the clustree package (version 0.5.1). By comparing one cluster to others using the FindAllMarkers function, we were able to locate marker genes in each cluster using the Wilcoxon rank-sum test. Based on SingleR annotation [17], manual annotation adjustment was carried out according to the cardinal marker genes of each cluster.

### Functional enrichment analysis

The hallmark gene sets were obtained from the Molecular Signature Database. The FindMarkers function in Seurat along with the defalut parameters allowed for the discovery of DEGs between two groups. The volcano plots were also made with the ggplot2 program. The DEGs were sorted by average log2 (fold change) following processing with a minimum log2 (fold change) of 0.5 and a maximum adjusted p value of 0.05. Using enrichGO and enrichKEGG in the clusterProfiler package (version 4.6.2), the functional enrichment analysis of Gene Ontology (GO) and Kyoto Encyclopedia of Genes and Genomes (KEGG) pathways words were performed. The GSVA package (version 1.46.0) was used to rate the pathway activity for every cell using gene set variation analysis (GSVA) methods [18]. And the GSEA analysis were also conducted by the ClusterProfiler package. The function of AddModuleScore in Seurat package was used to perform the functional gene set scores. The specific genes of the gene sets were referred to the study of Xuejing Sun et al. [19], which had removed the mouse-specific genes, and the list of specific genes was shown in **Additional file 2**. PPI analysis were conducted in the STRING website and visualized in Cytoscape software.

#### Cell-to-cell communication analysis

Cell-cell communication pathways were found using CellChat package (version 1.6.1) [20]. CellChat used permutation testing and a probability value system to extrapolate biologically significant cell-cell communication. Utilizing circle graphs, the communication network between several cell types were shown. The function of rankNet was used to compare the information flow of each signaling pathway to identify conservative and environment-specific signaling pathways. The signaling pathways were grouped based on functional similarity using joint manifold learning of the HC and STEMI communication networks. Based on the Euclidean distance in the learned joint manifold, signaling pathways that showed significant differences in functional similarity were found. The hierarchy diagram of the netVisual_aggregate function was used to display the specified signaling pathway. The function of netAnalysis_contribution was employed to display different receptor-ligand pairs under a given signaling pathway.

#### Quantitative real-time PCR (qRT-PCR)

For validation of the scRNA-seq findings, peripheral blood samples were collected from an independent cohort consisting of 20 STEMI patients and 8 healthy controls (HC) who were not included in the sequencing cohort. All participants were recruited from Fuwai Huazhong Cardiovascular Disease Hospital during the same study period and met identical inclusion and exclusion criteria as described above. Baseline demographic and clinical characteristics were comparable between the validation and sequencing cohorts, ensuring consistency and reliability of the results. The PrimeScript™ RT reagent Kit with gDNA Eraser for qPCR (Takara Biotech Inc., Shiga, Japan) was used to synthesize the first strand of cDNA. The cDNA was then amplified using TB Green^®^Premix Ex Taq™ II (Tli RNaseH Plus) (Takara Biotech Inc., Shiga, Japan) in accordance with the manufacturer’s instructions. The 2-^△△^Ct method was used to analyze the relative expression level of genes, which was computed using the internal control GAPDH. Below are the primers used in this study. JUN: Forward Primer: TCCAAGTGCCGAAAAAGGAAG; Reverse Primer: CGAGTTCTGAGCTTTCAAGGT; GAPDH: Forward Primer: GGAGCGAGATCCCTCCAAAAT; Reverse Primer: GGCTGTTGTCATACTTCTCATGG.

#### Statistical analysis

Using a two-tailed Wilcoxon rank sum test, the comparison of gene expression or signature between two groups or among clusters was confirmed. Using a two-tailed Student’s t-test, the cell distribution comparison between the two groups was confirmed. R (version 4.2.2) was used to express statistical analysis for single-cell data. GraphPad Prism 9 was used to analyze the expression of genes. It was deemed statistically significant when *P* < 0.05.

## Result

### The quality control of the study

Ten participants—seven STEMI cases and three controls—were examined in this study. The quality of PBMCs was assessed before the scRNA-seq was performed. The results of the cell count indicated that the average concentration of total cells was 1.97 × 10^6^ cell/ml, the average concentration of living cells was 1.84 × 10^6^ cell/ml, and the viability of PBMCs was 93.40%. These findings demonstrated that PBMCs quality satisfied the experimental prerequisites for scRNA-seq.

After quality control, transcriptome data comprising 29,723 cells with 26,980 genes in the HC group and 41,565 cells with 27,228 genes in the STEMI group were obtained. The quality controls for the 10 PBMC samples included assessment of gene count, UMI count, mitochondrial gene ratio, ribosomal gene ratio, and erythrocyte gene ratio. The original expression matrix, the matrix after removing doublets, and the matrix filtered according to quality control criteria were analyzed sequentially (Additional file 3. a-c). GO enrichment analysis of DEGs between the HC and STEMI groups revealed that most enrichment was associated with ribosome- and mitochondria-related functions (Additional file 3. d). Consequently, genes related to ribosomes, mitochondria, and erythrocytes were excluded in the further analysis (Additional file 3. e). The cell cycle scores of the included cells were near zero, indicating no significant impact from cell cycle-related genes (Additional file 3. f). These findings confirm that the expression matrix quality was adequate for subsequent analysis.

### Circulating immune cell profile in STEMI patients and HC

**Fig. 1:**
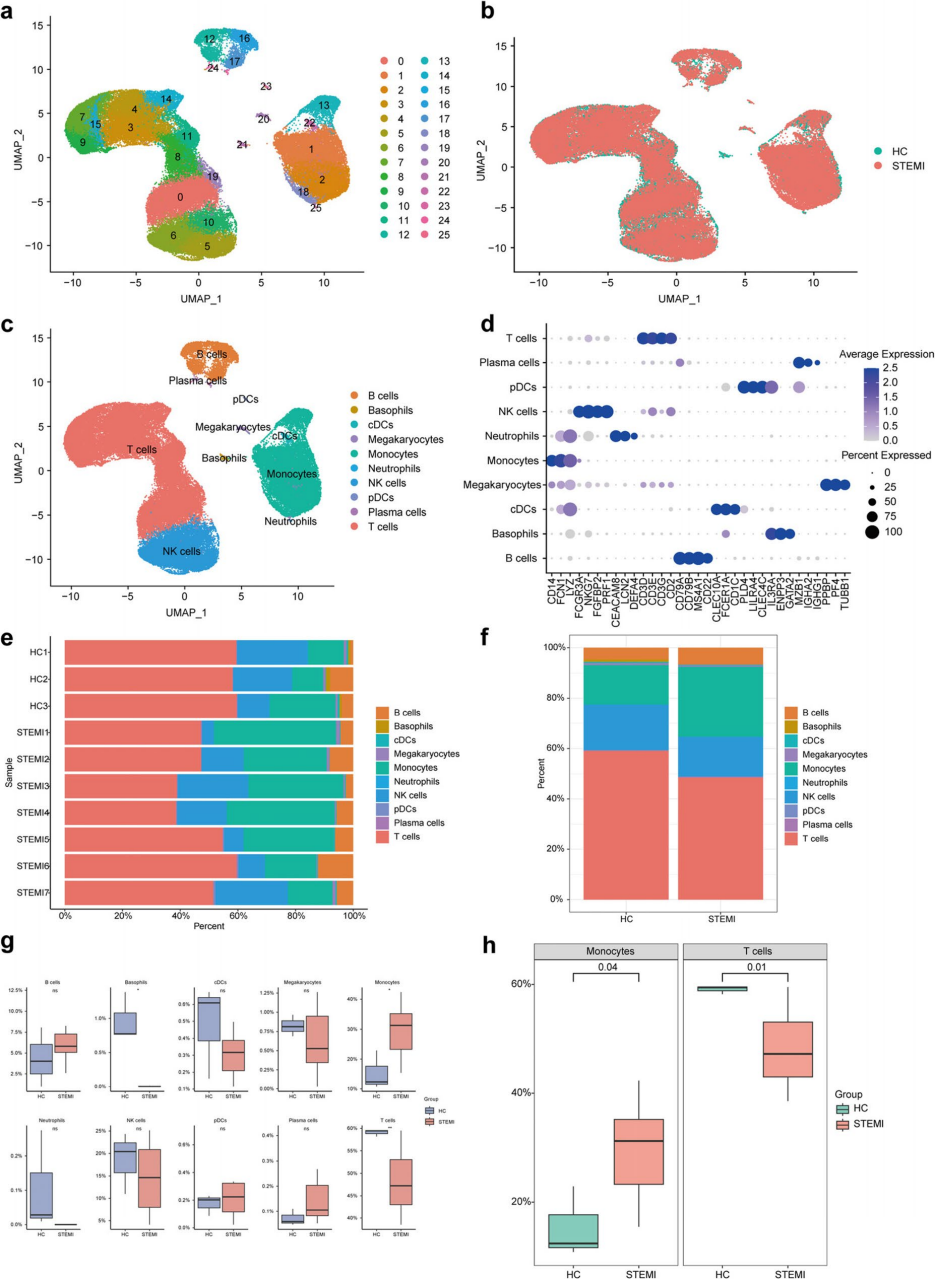
Circulating immune cell profile in STEMI patients and HC. **a** UMAP plot of all 71,288 PBMCs divided into 26 clusters from 7 STEMI patients and 3 HC participants. **b** UMAP plot of the distribution of PBMCs in STEMI and HC without batch effects. **c** Clusters of PBMCs were annotated into 10 cell types, including monocytes, T cells, NK cells, B cells, megakaryocytes, basophils, cDCs, pDCs, plasma cells and neutrophils. **d** Dot plot showed the marker genes of each cell type. **e** Percentage of each cell type in each sample for all 10 samples. **f** Bar plot showed the proportion of cell types in HC and STEMI groups. **g** Box plots comparing the proportion of each cell type between the two groups.The differences of monocytes and T cells were statistically significant. Two sides Student t-test was used, p value < 0.05. **h** Box plots of the comparison of the proportion in monocytes and T cells between the two groups, showing specific p value. Two sides Student t-test was used

After filtering, 71,288 cells in all were annotated. After dimension reduction by PCA, cells with approximate expression characteristics were clustered using the UMAP algorithm. The 10 PBMC samples were categorized into 26 clusters based on an unsupervised arrangement of gene expression analysis (Fig. 1a). The UMAP plot showed that the cells between the two groups were well integrated without batch effect **(**Fig. 1b**)**. The marker genes for each cluster were then examined. Based on marker gene annotation, ten cell types were identified from a total of 26 clusters: monocytes (clusters 1, 2, 13 and 18), T cells (clusters 0, 3, 4, 7, 8, 9, 11, 14, 15 and 19), NK cells (clusters 5, 6, and 10), B cells (clusters 12, 16 and 17), megakaryocytes (cluster 20), basophils (cluster 21), conventional dendritic cells (cDCs, clusters 22), plasmacytoid dendritic cells (pDCs, clusters 23), plasma cells (cluster 24) and neutrophils (cluster 25) (Fig. 1c). The validation of the annotation was achieved by the display of markers specific to distinct cell types (Fig. 1d). The percentage of cell types was calculated for each sample (Fig. 1e). The comparison of cell type proportions between the HC and STEMI group revealed variations in immune cell distribution (Fig. 1f). Statistical analysis further demonstrated significant differences in the percentages of basophils, monocytes, and T cells between the two groups (Fig. 1g). The p value for the statistical difference in monocytes and T cells between the two groups was 0.04 and 0.01, respectively (Fig. 1h). Due to the extremely low proportion of basophils in the STEMI group, further analysis of basophils was not considered. Therefore, we next focused on the analysis of monocytes and T cells in STEMI and HC, in terms of cellular and genetic dimensions.

### Re-clustering of monocytes revealed different composition of subclusters between STEMI and HC

**Fig. 2:**
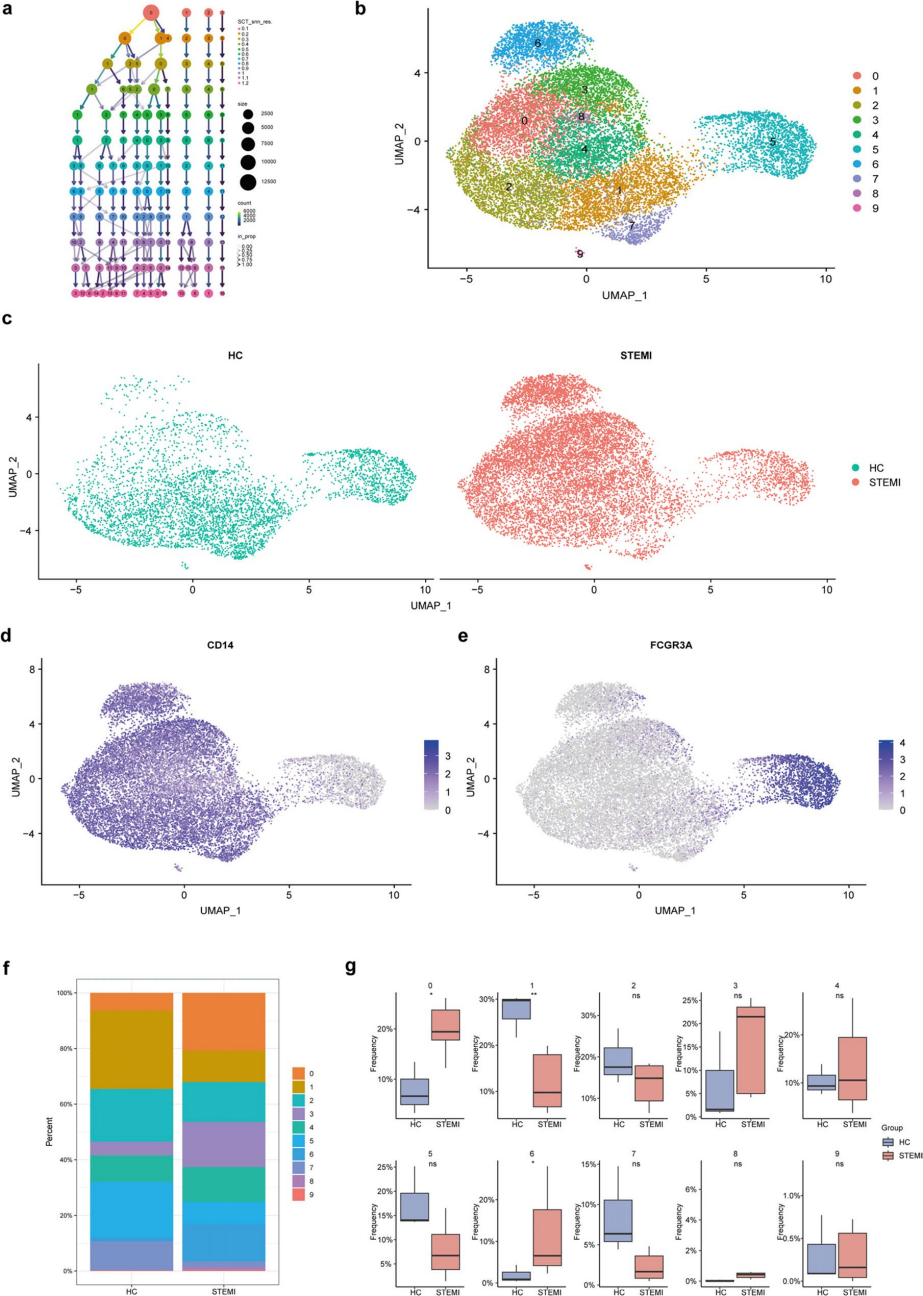
Re-clustering of monocytes revealed different composition of subclusters between STEMI and HC. **a** Tree plot showed the number of subclusters in monocytes when the resolution ranged from 0.1 to 1.2. **b** UMAP plot of 16,212 monocytes subdivided into 10 subclusters, with the resolution of 0.6. **c** UMAP plots of the distribution of 4658 and 11,554 monocytes in HC and STEMI. **d** Featue plot of CD14 in monocytes. **e** Featue plot of FCGR3A (CD16) in monocytes. **f** Bar plot showed the proportion of subclusters of monocytes in STEMI and HC groups. **g** Box plots comparing the proportion of each subclusters of monocytes between the two groups. The differences of subclusters 0, 1 and 6 were statistically significant. Two sides Student t-test was used, p value < 0.05. **h** Box plots of the comparison of the proportion in subclusters 0, 1 and 6 between the two groups, showing specific p value. Two sides Student t-test was used

We focused on monocytes for the follow-up analysis, extracting 16,212 monocytes from the overall expression matrix and performing re-clustering. The number of clusters obtained at different resolution settings ranging from 0.1 to 1.2 was evaluated (Fig. 2a). A resolution of 0.6 was selected, resulting in the categorization of monocytes into 10 subclusters (Fig. 2b). The distribution of 4658 and 11,554 monocytes in HC and STEMI was visualized (Fig. 2c). The expression levels of the marker genes CD14 and FCGR3A (CD16) in monocytes were presented, respectively (Fig. 2d and e). Traditionally, monocytes are classified into three subtypes based on the expression of CD14 and CD16; however, intermediate monocytes (CD14 + + CD16+) could not be distinguished in this analysis. Consequently, subsequent analysis was conducted based on the 10 identified clusters. The overall proportions of monocyte clusters in the HC and STEMI groups were compared (Fig. 2f), and differences in the proportions of each subcluster between the two groups were examined for statistical significance (Fig. 2g). The results indicated that clusters 0, 1, and 6 exhibited significant differences between the groups.

### Monocyte subclusters showed gene-level differences between STEMI and HC

**Fig. 3:**
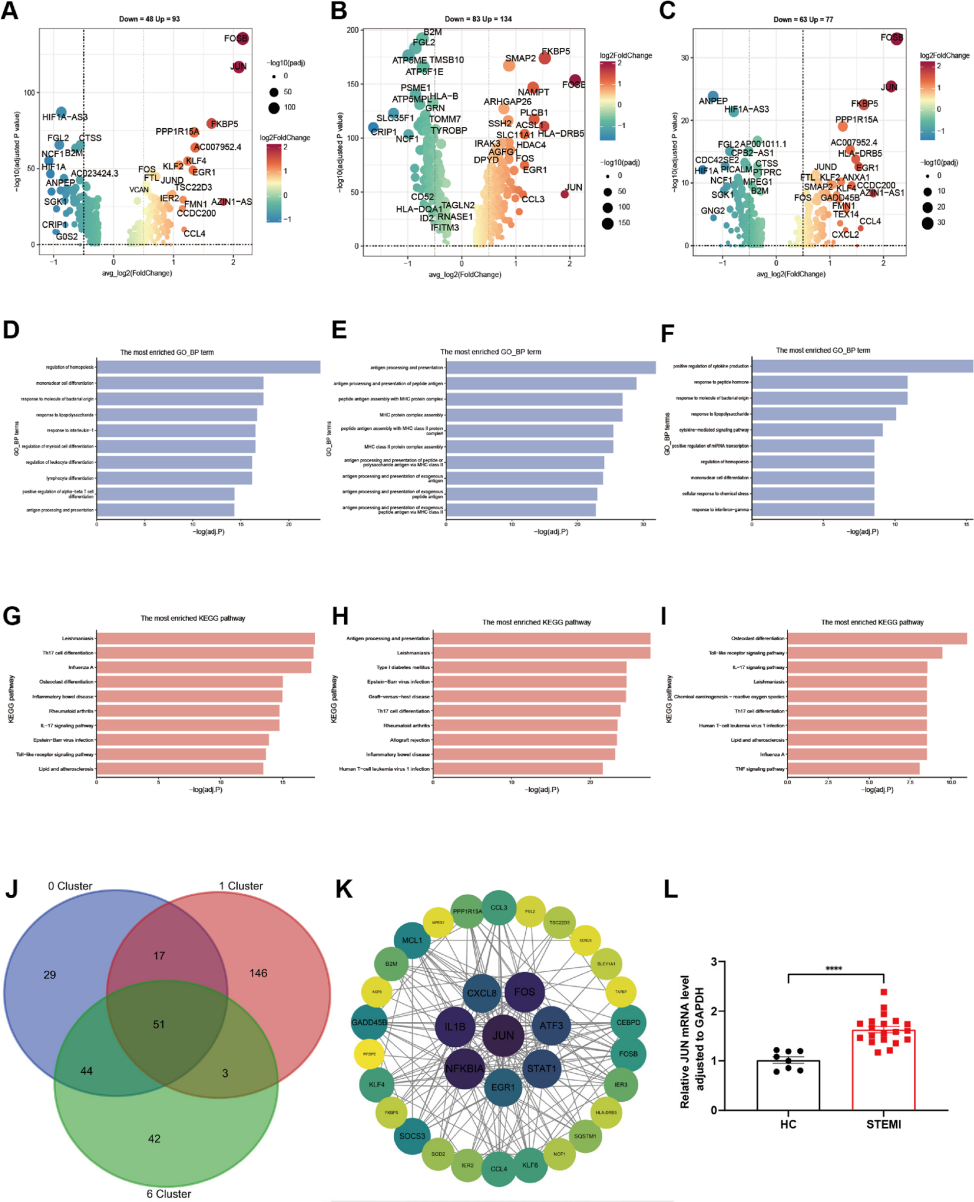
Monocyte subclusters showed gene-level differences between STEMI and HC. **a-c** Volcano plots showing DEGs in 3 subclusters: subcluster 0 (**a**), subcluster 1 (**b**), and subcluster 6 (**c**). Genes with significant upregulation (red) and downregulation (blue) are highlighted (|log2 fold change| >1, adjusted *p* < 0.05). Bubble size indicates the absolute log2 fold change, and color reflects adjusted p-value. **d-f** Bar plots showing the top enriched BP function in GO terms for the DEGs in subcluster 0 (**d**), subcluster 1 (**e**), and subcluster 6 (**f**). Terms are ranked by significance (-log10 adjusted p-value). **g-i** Bar plots showing the top enriched KEGG pathways for DEGs in subluster 0 (**g**), subluster 1 (**h**), and subluster 6 (**i**). Pathways are ranked by significance (-log10 adjusted p-value). **j** Venn diagram showing the overlap of DEGs among clusters 0, 1, and 6 (*n* = 51). **k** PPI network of the 51 shared genes based on STRING database analysis. Nodes represent proteins, and edges represent interactions. Key hub gene JUN are highlighted in purple, indicating the central regulatory role. **l** Validation of JUN expression by qRT-PCR in PBMCs from STEMI patients (*n* = 20) and HC participants (*n* = 8). Data are shown as mean ± SEM, normalized to GAPDH. Statistical analysis was performed using an unpaired two-tailed t-test. ****p < 0.0001

To identify critical genes involved in the development of STEMI and understand their unique roles, we analyzed the DEGs of subclusters 0, 1, and 6 in monocytes between STEMI and HC. The DEGs for each cluster were visualized using volcano plots (Fig. 3a-c). Detailed information on the top 10 DEGs in the three clusters between the two groups was provided (**Additional file 4**). Four DEGs—FOSB, JUN, FKBP5, and EGR1—were shared across subclusters 0, 1, and 6. Additionally, subclusters 0 and 6 shared three DEGs (AZIN1-AS1, CCL4, and AC007952.4), while FKBP5 was shared between subclusters 1 and 6.

To further explore the functional roles of these DEGs in monocytes, GO and KEGG enrichment analyses were performed for the 3 subclusters. The top 5 GO terms for biological processes (BP), cellular components (CC), and molecular functions (MF) were summarized (Additional file 5. a-c). Focusing on BP functions, the top 10 enriched terms were identified for subclusters 0 (inflammatory-like), 1 (antigen-presenting), and 6 (cytokine-regulatory) (Fig. 3d-f). For subcluster 0, the significantly enriched genes were primarily associated with inflammatory responses, such as response to molecules of bacterial origin, response to lipopolysaccharide, and response to interleukin-1 (Fig. 3d). In subcluster 1, genes with significant differences between the two groups were mainly related to immune responses, such as antigen processing and presentation and peptide antigen assembly with MHC protein complexes (Fig. 3e). In subcluster 6, the significantly enriched genes were primarily involved in cytokine regulation, such as positive regulation of cytokine production and cytokine-mediated signaling pathways (Fig. 3f). KEGG enrichment analysis revealed that DEGs in all 3 subclusters were significantly enriched in pathways such as Th17 cell differentiation, the IL-17 signaling pathway, and inflammatory bowel disease, which are closely related to immunity and inflammation (Fig. 3g-i).

There were 51 genes in the overlap of DEGs across subclusters 0, 1, and 6 (Fig. 3j). PPI analysis of these 51 genes identified JUN as the key hub gene (Fig. 3k). To validate the expression of the JUN gene in PBMCs of STEMI patients and HC, qPCR was performed on samples from 20 STEMI patients and 8 HC participants. The relative mRNA expression of JUN, normalized to GAPDH, was significantly higher in STEMI patients (1.63 ± 0.29) compared to HC (1.02 ± 0.18), with a p-value of < 0.0001 (Fig. 3l). This result confirmed that the expression of JUN in PBMCs is significantly upregulated in STEMI patients compared to healthy controls.

### Re-clustering of T cells revealed different composition of subclusters between STEMI and HC

**Fig. 4:**
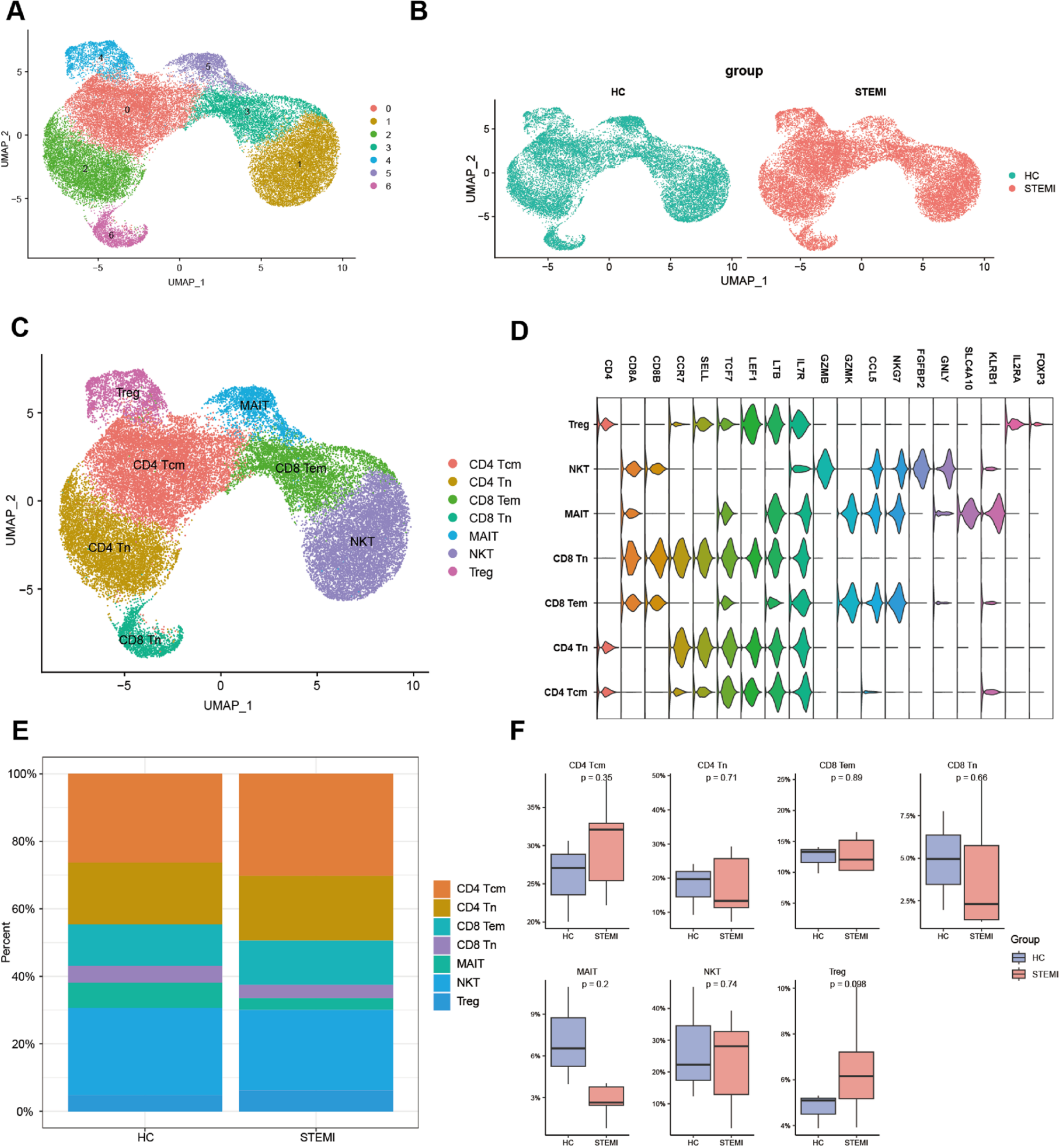
Re-clustering of T cells revealed different composition of subclusters between STEMI and HC. **a** UMAP plot of 37,704 T cells subdivided into 10 subclusters. **b** UMAP plots of the distribution of 17,551 and 20,153 T cells in HC and STEMI. **c** UMAP plot of T cells annotated into 7 cell types, including CD4 Tcm, CD4 Tn, CD8 Tem, CD8 Tn, MAIT, NKT and Treg. **d** Violin plots showed the marker genes of subtypes of T cells. **e** Bar plot showed the proportion of subtypes of T cells in HC and STEMI groups. **f** Box plots comparing the proportion of each subtypes of T cells between the two groups. Two sides Student t-test was used, *p* value < 0.05

The proportion of T cells in PBMCs was the largest among cell types, with a significant difference observed between the HC and STEMI groups, prompting further analysis. A total of 37,704 T cells were extracted from the overall expression matrix and re-clustered into 7 subclusters (Fig. 4a). Among these, 17,551 T cells were from the HC group and 20,153 from the STEMI group (Fig. 4b). The T cells were classified into seven subtypes: CD4-positive central memory T cells (CD4 Tcm), CD4-positive naive T cells (CD4 Tn), CD8-positive effector memory T cells (CD8 Tem), CD8-positive naive T cells (CD8 Tn), mucosal-associated invariant T cells (MAIT), natural killer T cells (NKT), and regulatory T cells (Treg) (Fig. 4c). The marker genes for each T cell subtype were identified (Fig. 4d). A comparison of the proportions of the seven T cell subtypes between the two groups showed no statistically significant differences, as all p-values were greater than 0.05 (Fig. 4e and f).

### T cell subclusters showed gene-level differences between STEMI and HC

**Fig. 5:**
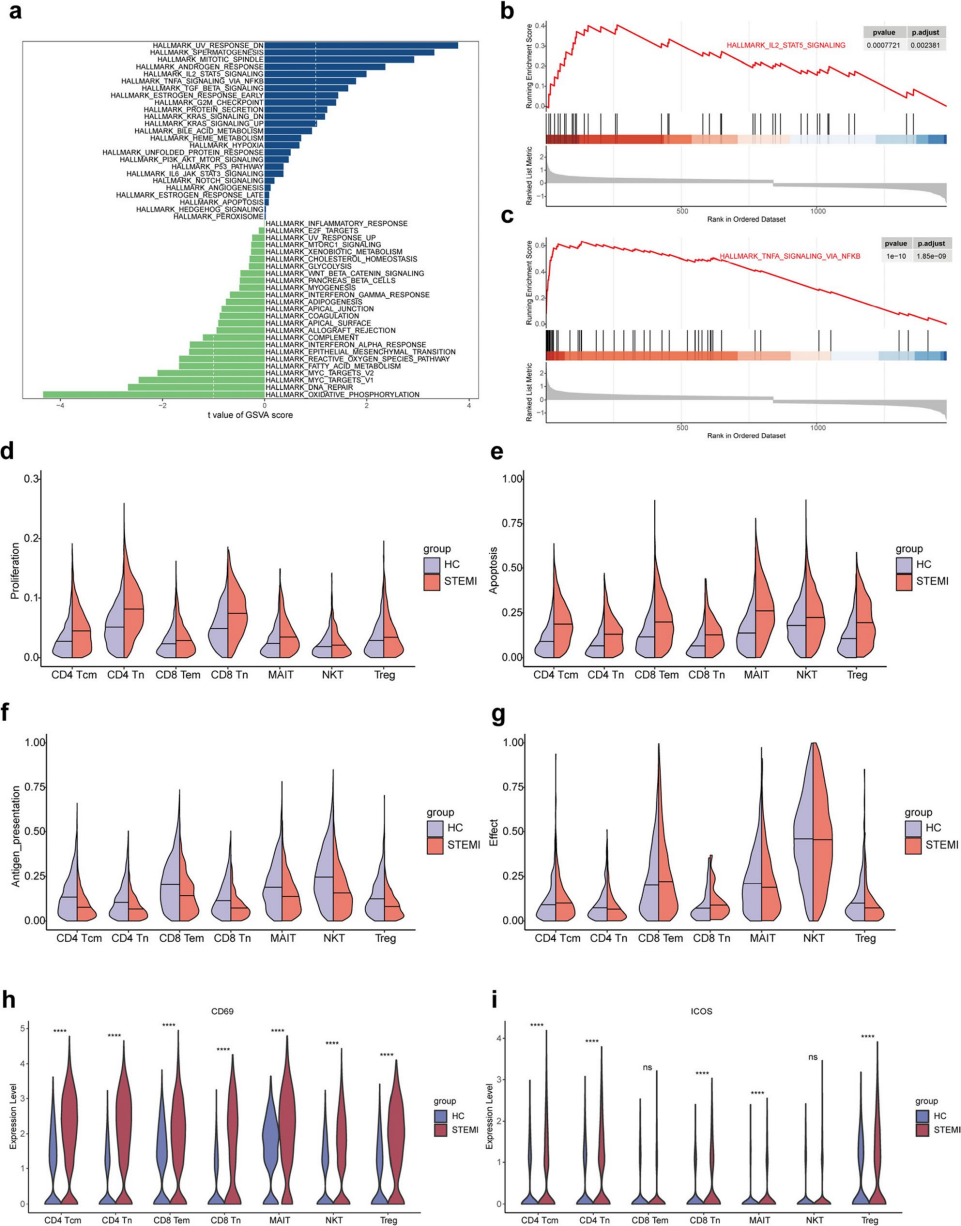
T cell subclusters showed gene-level differences between STEMI and HC. **a** Bar plot of GSVA showing significantly upregulated (blue) and downregulated (green) hallmark pathways in STEMI patients compared to HC. The value in the bottom represents the difference between the t value of a gene set in the STEMI and HC. **b and c** GSEA for hallmark pathways. **b** The “IL2-STAT5 signaling” pathway is significantly upregulated in STEMI patients. **c** The “TNF-α signaling via NF-κB” pathway is also significantly enriched in STEMI patients. **d-g** Violin plots comparing key immune functional gene sets scores between STEMI patients and HC across subclusters. The functional gene sets scores include proliferation (**d**), apoptosis (**e**), antigen presentation (**f**), and effector (**g**). **h and i** Violin plots depicting the expression levels of key immune activation markers CD69 (**h**) and ICOS (**i**), across T cell subclusters. Statistical significance is indicated as follows: ****p < 0.0001, ***p < 0.001, **p < 0.01, *p < 0.05

Subsequently, gene-level analyses were performed on T cells. GSVA was conducted using the T cell expression matrix for the 50 gene sets from the Hallmark database. The enrichment scores for each gene set across samples in the two groups were displayed (Additional file 6). Differential analysis of GSVA scores between the groups revealed significant differences in the inflammation-related gene sets IL2-STAT5 signaling and TNF-α signaling via NF-κB, which were enriched in the STEMI group (Fig. 5a). As shown in Additional file 6, the IL2-STAT5 signaling was enriched in six of seven STEMI patients, whereas no enrichment was observed in the healthy control group, indicating that this activation pattern was consistent across most STEMI patients. GSEA of these two gene sets in the DEGs of T cells showed positive enrichment with ES peaking at the front end and very small p-values and adjusted p-values (Fig. 5b **and c**). It indicated that the genes in these inflammation-related gene sets were significantly enriched in the STEMI group.

Key immune gene sets related to proliferation, apoptosis, antigen presentation, and effector functions were further analyzed in T cell subtypes. The enrichment scores of these gene sets showed that the proliferation and apoptosis gene sets had higher scores in the STEMI group compared to the HC group, while the antigen presentation gene set score was slightly lower in the STEMI group. The effector function gene set scores were comparable between the two groups (Fig. 5d, e, f and g). These findings suggest that STEMI patients exhibit altered immune functions, such as dysregulated apoptosis. The expression levels of CD69 and ICOS, two key markers of T cell activation, were demonstrated using violin plots (Fig. 5h and i). The expression of CD69 was significantly higher in all T cell subclusters in the STEMI group compared to the HC group, indicating that T cells were significantly activated in the STEMI group.

### Changes in intercellular communication of PBMCs in STEMI

**Fig. 6:**
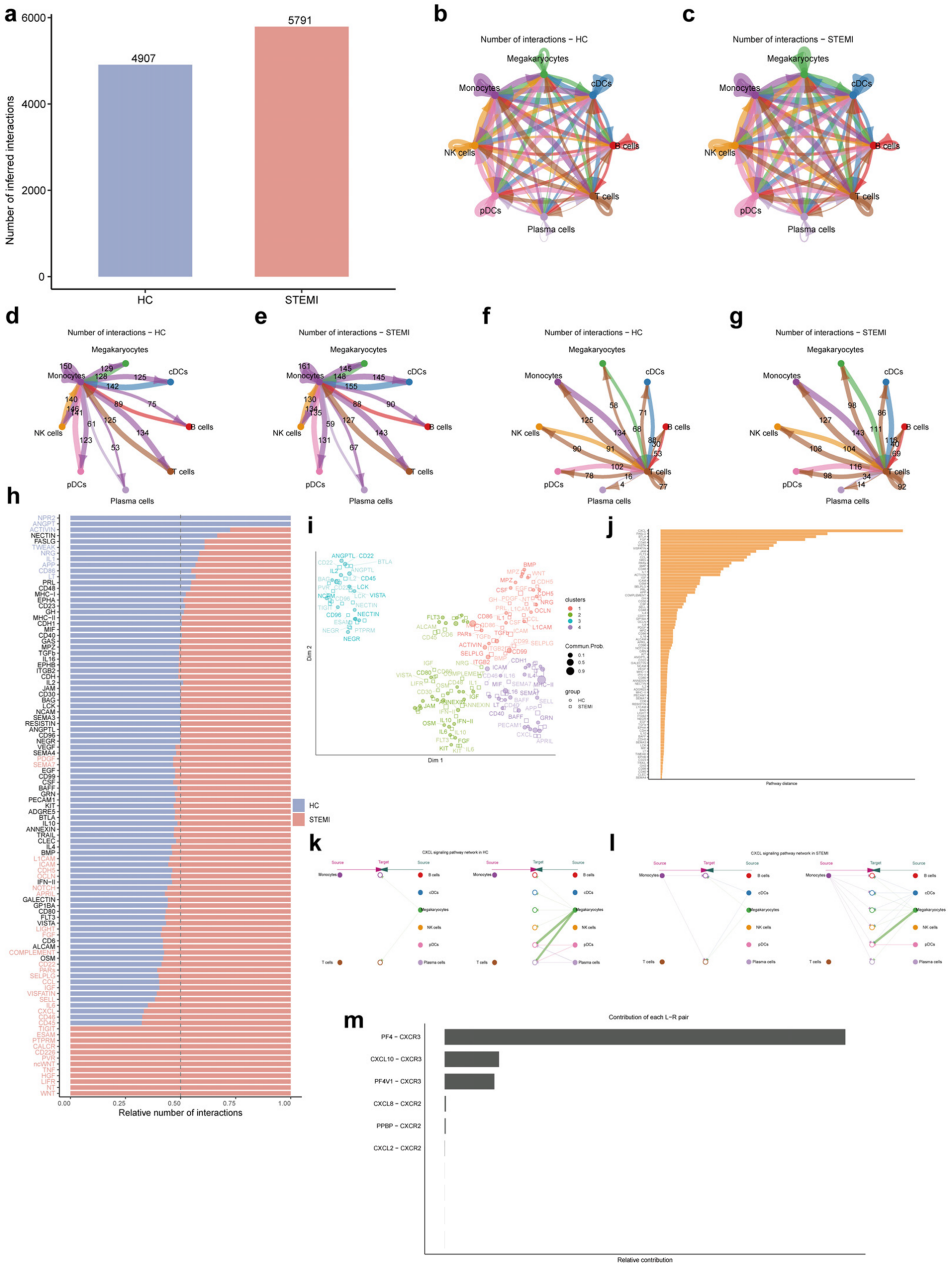
Changes in intercellular communication of PBMCs in STEMI. **a** Bar plot showing the total number of inferred intercellular interactions in PBMCs from STEMI patients and HC. **b** and **c** Circle plots illustrating the overall intercellular interaction networks for HC (**b**) and STEMI (**c**). Line thickness indicates the number of interactions between cell types, showing elevated intercellular communication in STEMI. **d** and **e** The communication network focusing on the interactions between monocytes and other cell types in HC (**d**) and STEMI (**e**). **f** and **g** The communication network focusing on the interactions between T cells and other cell types in HC (**f**) and STEMI (**g**). **h** Bar plot showing the important signaling pathways based on the difference in the overall signal flow in the inferred network between STEMI and HC. **i** UMAP plot shows the signaling pathways were grouped based on functional similarity. **j** Bar plot ranking the frequency of ligand-receptor interactions, with CXCL signaling identified as a top pathway. **k-l** Hierarchy plots of CXCL signaling pathways in HC (**k**) and STEMI (**l**). **m** Bar plot showing the contribution of key ligand-receptor pairs in the CXCL signaling pathway in STEMI, with PF4-CXCR3 contributing the most

CellChat was employed to evaluate cell-cell communication networks by inferring potential receptor-ligand interactions based on single-cell gene expression data. Using this approach, the intercellular communication network among different PBMC cell types was investigated. The total number of inferred intercellular interactions was found to be increased in the STEMI group, indicating enhanced intercellular communication in the disease state (Fig. 6a). The circle networks illustrating the number of intercellular interactions in the HC group and STEMI group were visualized separately (Fig. 6b and c). Due to their low cell numbers (< 10) in the STEMI group, neutrophils and basophils were excluded from the analysis.

The communication network of monocytes with other cell types was analyzed, revealing that monocytes in the STEMI group engaged in a greater number of interactions with the remaining 7 cell types compared to the HC group (Fig. 6d and e). Similarly, T cell interactions with the other 7 cell types were more frequent in the STEMI group than in the HC group (Fig. 6f and g). To identify conserved and environment-specific signaling pathways, the information flow of each signaling pathway was compared. Information flow, defined as the sum of communication probabilities between all cell pairs in the inferred network, showed notable differences between the STEMI and HC groups, with important signaling pathways ranked based on the differences in signal flow (Fig. 6h). Longer purple bars indicated greater enrichment in the HC group, while longer light red bars reflected greater enrichment in the STEMI group. Signaling pathways were grouped by functional similarity, determined by the similarity between sender and receiver cells (Fig. 6i). Based on the Euclidean distance of the signal network in a shared two-dimensional space, the CXCL pathway emerged as the most divergent signaling pathway, demonstrating the greatest difference in functional similarity of the communication network between the STEMI and HC groups (Fig. 6j). The CXCL signaling pathway was visualized using hierarchy plots for the STEMI and HC groups (Fig. 6k and l). In both groups, the left part of the plot showed monocytes and T cells as signaling receivers, with increased autocrine and paracrine signaling by monocytes in the STEMI group. The right part depicted monocytes and T cells as signaling senders, highlighting enhanced paracrine signaling by monocytes in the STEMI group. Furthermore, PF4-CXCR3 was identified as the most contributory ligand-receptor pair in the CXCL signaling network of PBMCs from STEMI patients (Fig. 6m).

## Discussion

In this study, we characterized the transcriptome profiles of PBMCs from STEMI patients and HC participants at the single-cell level, focusing on elucidating the subpopulation composition of monocytes and T cells, gene expression levels, major enriched gene sets and cellular communication among different cell types. The key hub gene of monocytes in PBMCs was identified and validated, and the expression of T cell activation genes as well as signaling pathways highly expressed in STEMI patients in the cellular communication network were observed.

In order to describe the transcriptome profile of PBMCs in STEMI patients and HC participants, we collected peripheral blood from 10 subjects − 7 STEMI patients and 3 HC participants, aged 50–75 years. There was an imbalance between the number of STEMI patients (*n* = 7) and healthy controls (*n* = 3), which mainly resulted from the limited availability of high-quality PBMC samples that passed stringent quality control for sequencing. To minimize the potential bias introduced by this imbalance, we performed rigorous batch correction and data integration using the Harmony algorithm, which effectively aligned cells across individuals and experimental conditions. Importantly, the cellular and transcriptional alterations observed in STEMI patients were highly consistent across individuals, supporting the robustness of our findings.In previous studies comparing PBMCs STEMI patients with healthy individual [22], the age criterion for enrollment was more than 18 years, including both young and elderly people. However, it is not reasonable to consider patients with early-onset coronary artery disease who developed STEMI at less than 45 years old together with the elderly [23, 24]. In our study, the age range was narrower, focusing on patients with high prevalence of coronary heart disease. After the collection of peripheral blood, the isolation was performed to obtain PBMCs and 71,288 cells and 28,118 genes were finally analyzed after library construction and quality control. These PBMCs were divided into 26 clusters after dimensionality reduction and clustering, and were categorized into 10 cell types after cellular annotation, including Monocytes, T cells, NK cells, B cells, megakaryocytes, basophils, cDCs, pDCs, plasma cells and neutrophils. The marker genes used for cellular annotation were specifically expressed in different cell types, which was consistent with the other literature [25, 26].

Current studies have shown that monocytes play a key role in AMI and atherosclerosis, and that targeting peripherally circulating monocytes may be crucial for the acute management of STEMI. By intervening monocytes, the occurrence and development of STEMI complications can be prevented and reduced [27, 28]. Compared with HC, the number of monocytes in STEMI patients was significantly increased, which was consistent with the study of Irene V. van Blokland et al. [22]. Monocytes exhibit significant variety, which have been represented in a nomenclature that differentiates among CD14 + + CD16- classical, CD14 + + CD16 + intermediate, and CD14 + CD16 + + non-classical monocytes based on the expression of CD14 and CD16 (FCGR3A) [21]. Now, High-parameter cytometry and scRNA-seq have allowed for the emergence of a monocyte heterogeneity paradigm that goes beyond the present tripartite subset division [29]. In our study, there were 16,212 monocytes were redivided into 10 subclusters. Among these subclusters, three subclusters with significant differences in number between the two groups were selected for genetic analysis. Among them, the DEGs with significant differences between the two groups of subcluster 0 were mainly related to inflammatory responses, the DEGs of subcluster 1 were mainly related to immune responses such as antigen presentation, and the DEGs of subcluster 6 were mainly involved in cytokine regulation. And the key hub gene-JUN, which was one of the overlap genes of DEGs between these three subclusters, was identified and the expression of JUN was verified in PBMCs of STEMI patients and HC. JUN expression was higher in PBMCs of STEMI patients than in HC. According to previous research, the c-Jun/AP-1 signaling pathway may have been connected to vascular tissue healing following intravascular stent insertion [30] as well as coronary heart disease [31]. Previous studies have also demonstrated that pharmacologic inhibition of JNK, the upstream activator of the c-Jun/AP-1 pathway, mitigates myocardial injury and remodeling following infarction [32]. In this context, the upregulation of JUN observed in our study reinforces the importance of the JNK–c-Jun/AP-1 axis in post-infarction inflammation and repair. Thus, JUN may serve as a potential transcriptional indicator or therapeutic co-target within this established signaling pathway. Our findings provide additional evidence linking this pathway to the pathophysiology of STEMI and may guide future strategies aimed at modulating inflammation and tissue recovery.

Another cell type with significant differences in number was T cells, which were divided into 7 subclusters after re-clustering. Combined with T cell marker genes, T cells were named CD4 Tcm, CD4 Tn, CD8 Tem, CD8 Tn, MAIT, NKT and Treg according to their functional status. There was no significant difference in the number of these subtypes between the groups. It has been shown that there was a decrease in CD8 + cells after reperfusion [33], and in our study, the number of cells in CD8 Tem, CD8Tn and MAIT were also all lower than that in healthy controls, although the difference between groups was not significant. GSEA analysis of T cells showed that genes in the IL2-STAT5 signaling and TNF-α signaling via NF-κB pathways were enriched in STEMI patients. Interestingly, the IL2–STAT5 signaling pathway plays a central role in the activation and expansion of Tregs, which are critical for limiting post-infarction inflammation. Previous clinical studies of low-dose IL-2 therapy in patients with ischemic heart disease have demonstrated enhanced Treg responses and cardioprotective effects [34, 35]. Therefore, the activation of IL2–STAT5 signaling observed in our dataset may reflect an endogenous immune regulatory process, consistent with the mechanisms targeted by IL-2–based immunotherapy after myocardial infarction. Similarly, infliximab, a TNF-α inhibitor, has been studied to improve inflammation AMI induced in a porcine myocardial infarction model [36]. Proliferation, apoptosis, antigen presentation, and effect are important functions of T cells. There was no significant difference in gene set scores between the two groups. But the expression of T cell activation-related genes, CD69 [37] and ICOS [38], are significantly increased in STEMI, which may be due to the fact that the time of blood collection was within 6 h. T cells in the early stage was not yet been fully utilized, but it has begun to respond to the changes caused by STEMI. Studies have shown that the Annexin-A1 mimetic RTP-026 can promote cardiac protection by regulating immune cell activation [39].Our study also provided evidence that the prognosis of STEMI can be improved from the perspective of regulating T cell activation. In the future, blood samples can be collected in different time points to focus on the dynamic changes of T cells, and can be combined with heart tissue samples at the same time point to determine whether some transcriptomic changes occur earlier in peripheral blood, as in monocytes [40].

In the analysis of intercellular communication among different cell types of PBMCs, it was found that the number of intercellular communication in monocytes and T cells was increased in STEMI patients, although the cell number of T cells in STEMI patients was lower than that in HC. It was determined that the CXCL signaling pathway differed most between the two groups, and the probability of cell communication was higher in STEMI. Both paracrine and autocrine of CXCL signaling pathway in monocytes increased in STEMI patients, among which PF4-CXCR3 ligand receptor contributed the most. PF4 is a platelet-derived chemokine known to bridge thrombosis and inflammation by promoting monocyte recruitment and platelet–leukocyte interactions. Such platelet–immune crosstalk is a hallmark of thromboinflammation, which critically determines myocardial injury and repair after infarction [41–43]. Therefore, the upregulation of PF4–CXCR3 signaling observed in our study may reflect enhanced thromboinflammatory activity in STEMI, highlighting chemokine pathways as potential therapeutic targets.

From a translational perspective, JUN and CXCL signaling pathways identified in our study may intersect with current anti-inflammatory therapeutic strategies for STEMI. The JNK–c-Jun/AP-1 axis integrates inflammatory and stress signals, and pharmacologic inhibition of JNK has shown cardioprotective effects in experimental AMI models. Likewise, the PF4–CXCR3 chemokine pathway links platelet activation with immune-driven thromboinflammation, suggesting that modulation of these pathways may complement existing interventions such as IL-1β blockade and IL-2-based immunotherapy. Consistent with the findings of van Blokland et al. [22], our analysis also revealed pronounced alterations in monocyte and T-cell subsets in STEMI patients compared with healthy controls. Notably, both studies identified inflammatory activation and interferon-related transcriptional programs as key immune signatures following acute myocardial infarction. Our results further extend these observations by delineating IL2–STAT5 and CXCL signaling as additional immune-regulatory pathways potentially involved in post-infarction remodeling, providing complementary insights into the immune landscape of STEMI.

This study has several limitations. First, the sample size was relatively small due to the high cost of single-cell RNA sequencing, and there was an imbalance between the STEMI (*n* = 7) and healthy control (*n* = 3) groups, which may introduce potential bias. Second, only male participants were included, which limits the generalizability of the findings to female patients. Third, the PBMC samples were collected within 6 h after PPCI, representing the early phase of STEMI, and thus may not fully capture the dynamic immune changes that occur during later recovery stages. Finally, this study lacks experimental validation for the identified immune cell subsets and signaling pathways. Future studies with larger, more balanced cohorts and complementary experimental approaches, such as flow cytometry, qPCR, or immunofluorescence, are warranted to confirm these findings and further elucidate the immune mechanisms underlying STEMI.

## Conclusion

Single-cell transcriptomic analysis of PBMCs from STEMI patients revealed significant alterations in immune cell composition and transcriptional profiles, highlighting the key roles of monocytes and T cells in post-infarction inflammation. These findings provide novel insights into the immune mechanisms of STEMI and may inform future strategies for anti-inflammatory therapy.

## Supplementary Information

Supplementary Material 1: Online Resource 1 Clinical data of the 7 STEMI patients participants in this study. Online Resource 2 The specific genes of the gene sets. Online Resource 3 The quality control of expression matrix by scRNA-seq. a-c The violin plots show gene count, UMI count, mitochondrial gene ratio, ribosomal gene ratio, and erythrocyte gene ratio for the original expression matrix(a), the matrix after removing doublets(b), and the matrix filtered according to quality control criteria(c). d The dot plot of GO enrichment analysis of DEGs between the HC and STEMI groups. e The violon plots for the expression matrix after removing genes related to ribosomes, mitochondria, and erythrocytes. f The cell cycle scores for the expression matrix. Online Resource 4 The DEGs of subclusters 0, 1 and 6 of monocytes between STEMI and HC (top 10). Online Resource 5 a-c The top 5 GO enrichment terms of BP, CC and MF for the DEGs in subclusters 0 (a), 1 (b) and 6 (c) in monocytes. Online Resource 6 The heatmap of the GSVA scores in the 50 gene sets from the Hallmark database for T cells across samples in HC and STEMI groups.

## Data Availability

The data that support the findings of this study are available from the corresponding author upon reasonable request.

## Abbreviations

STEMI: ST-segment elevation myocardial infarction
AMI: Acute myocardial infarction
PPCI: Primary percutaneous coronary intervention
PBMCs: Peripheral blood mononuclear cells
scRNA-seq: Single-cell RNA sequencing
DEGs: Differentially expressed genes
HC: Healthy controls
GEMs: Gel bead-in-emulsions
UMI: Unique molecular identifier
UMAP: Uniform Manifold Approximation and Projection
GO: Gene Ontology
KEGG: Kyoto Encyclopedia of Genes and Genomes
PPI: Protein-protein interactions
