# Supplementary figures and images for: Single-cell transcriptomic analysis of peripheral blood mononuclear cells reveals key immune responses in ST-segment elevation myocardial infarction
Source: Hereditas. 2025 Nov 25;162:237. doi: 10.1186/s41065-025-00609-y (PMC12742203; doi:10.1186/s41065-025-00609-y)

**a**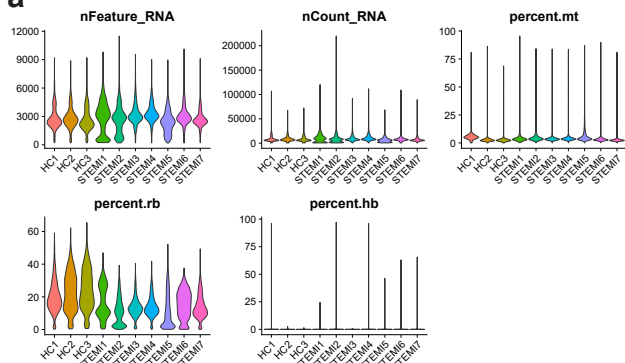**b**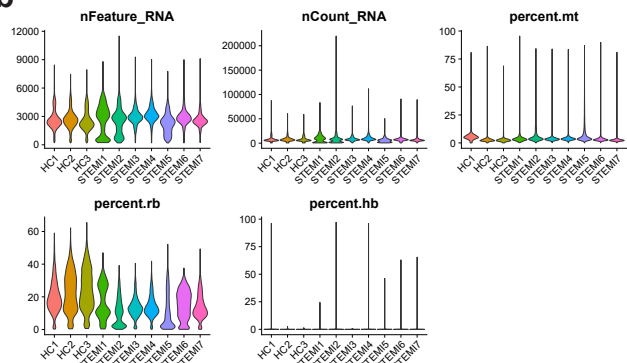**c**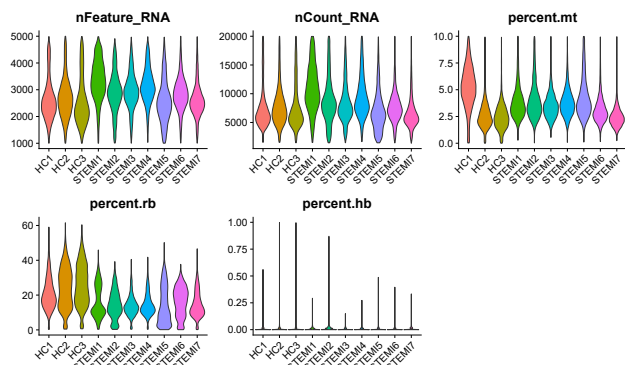**d**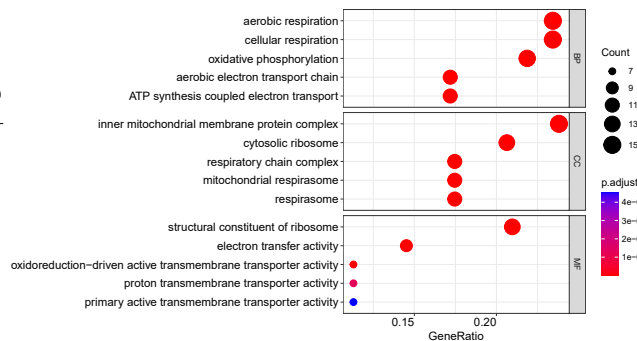**e**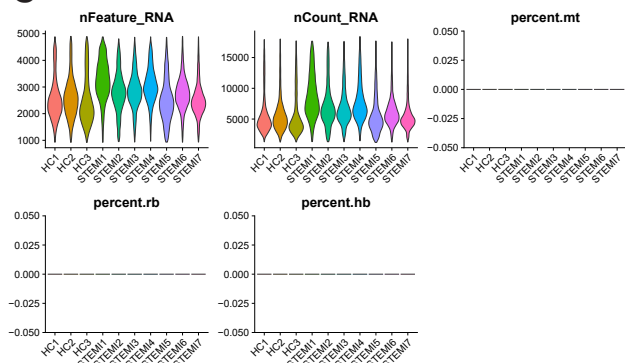**f**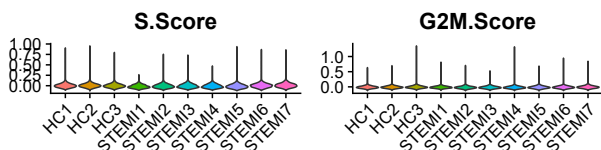

Supplement: Supplementary file 1 — Supplementary Material 1: Online Resource 1 Clinical data of the 7 STEMI patients participants in this study. Online Resource 2 The specific genes of the gene sets. Online Resource 3 The quality control of expression matrix by scRNA-seq. a-c The violin plots show gene count, UMI count, mitochondrial gene ratio, ribosomal gene ratio, and erythrocyte gene ratio for the original expression matrix(a), the matrix after removing doublets(b), and the matrix filtered according to quality control criteria(c). d The dot plot of GO enrichment analysis of DEGs between the HC and STEMI groups. e The violon plots for the expression matrix after removing genes related to ribosomes, mitochondria, and erythrocytes. f The cell cycle scores for the expression matrix. Online Resource 4 The DEGs of subclusters 0, 1 and 6 of monocytes between STEMI and HC (top 10). Online Resource 5 a-c The top 5 GO enrichment terms of BP, CC and MF for the DEGs in subclusters 0 (a), 1 (b) and 6 (c) in monocytes. Online Resource 6 The heatmap of the GSVA scores in the 50 gene sets from the Hallmark database for T cells across samples in HC and STEMI groups. [file 41065_2025_609_MOESM1_ESM.zip › Additional file 1-6/Additional file 3.pdf]

**a**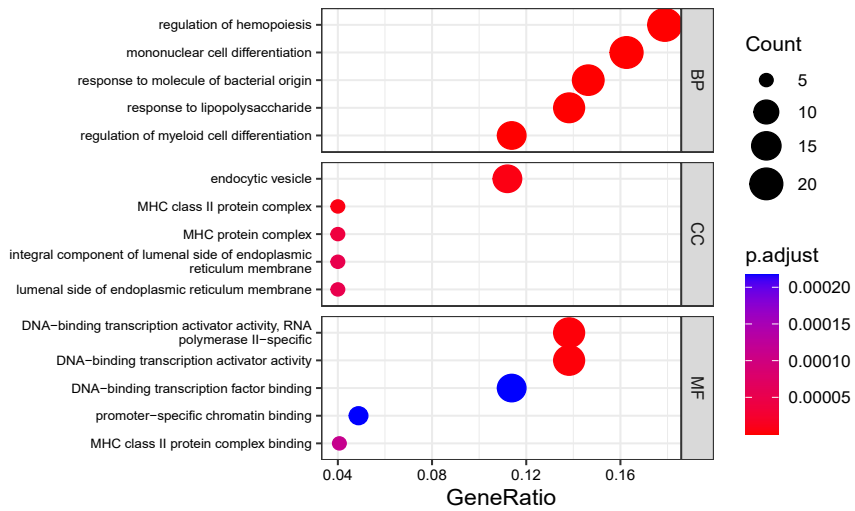**b**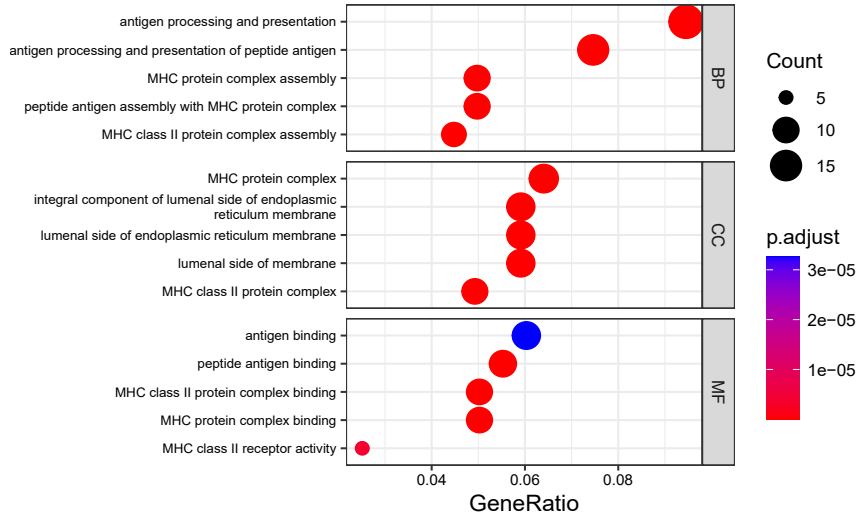**c**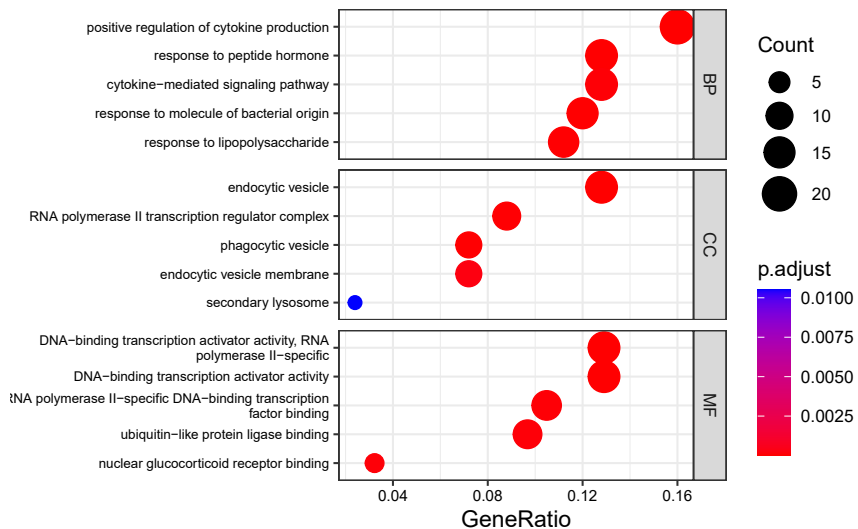

Supplement: Supplementary file 1 — Supplementary Material 1: Online Resource 1 Clinical data of the 7 STEMI patients participants in this study. Online Resource 2 The specific genes of the gene sets. Online Resource 3 The quality control of expression matrix by scRNA-seq. a-c The violin plots show gene count, UMI count, mitochondrial gene ratio, ribosomal gene ratio, and erythrocyte gene ratio for the original expression matrix(a), the matrix after removing doublets(b), and the matrix filtered according to quality control criteria(c). d The dot plot of GO enrichment analysis of DEGs between the HC and STEMI groups. e The violon plots for the expression matrix after removing genes related to ribosomes, mitochondria, and erythrocytes. f The cell cycle scores for the expression matrix. Online Resource 4 The DEGs of subclusters 0, 1 and 6 of monocytes between STEMI and HC (top 10). Online Resource 5 a-c The top 5 GO enrichment terms of BP, CC and MF for the DEGs in subclusters 0 (a), 1 (b) and 6 (c) in monocytes. Online Resource 6 The heatmap of the GSVA scores in the 50 gene sets from the Hallmark database for T cells across samples in HC and STEMI groups. [file 41065_2025_609_MOESM1_ESM.zip › Additional file 1-6/Additional file 5.pdf]

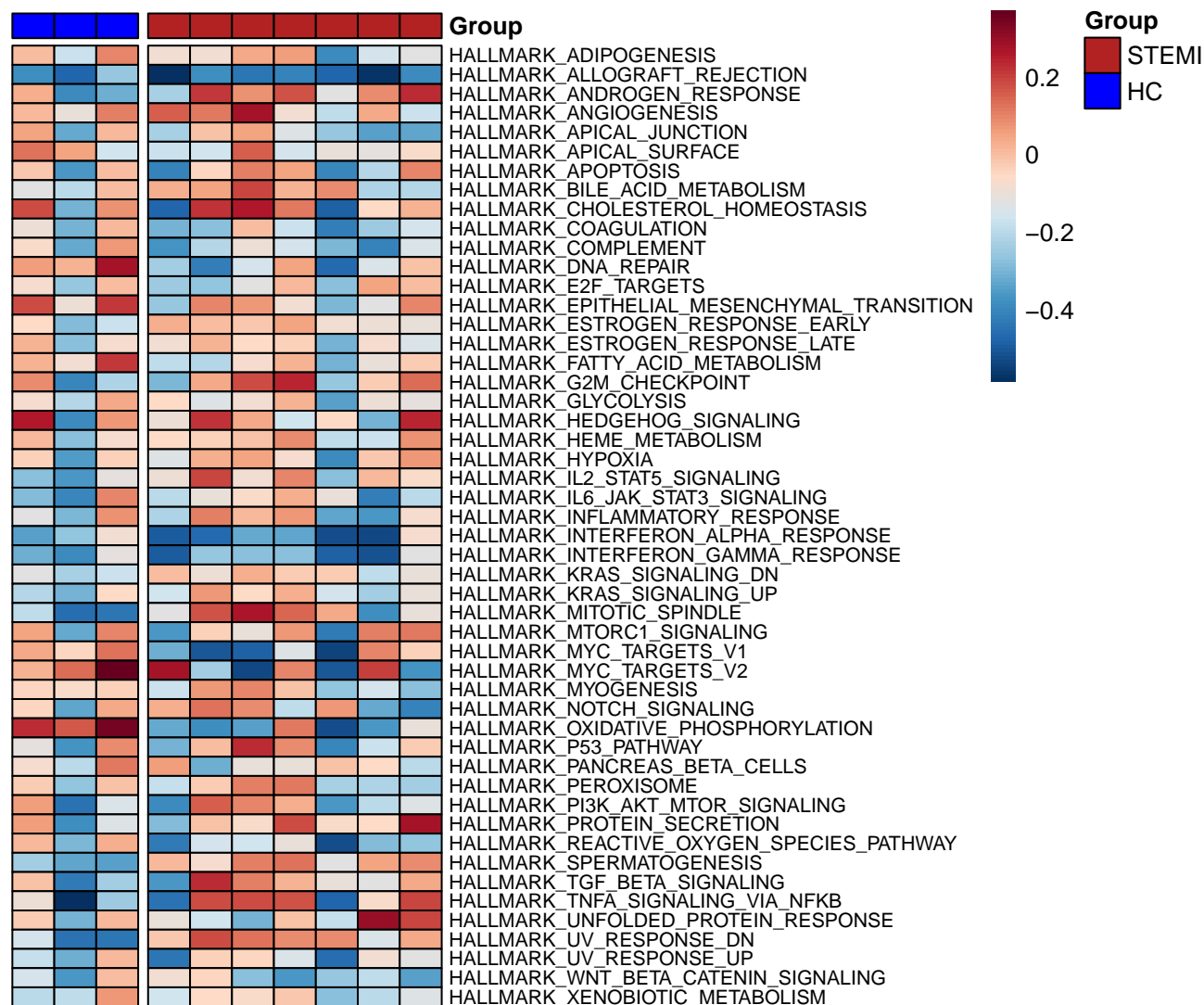

Supplement: Supplementary file 1 — Supplementary Material 1: Online Resource 1 Clinical data of the 7 STEMI patients participants in this study. Online Resource 2 The specific genes of the gene sets. Online Resource 3 The quality control of expression matrix by scRNA-seq. a-c The violin plots show gene count, UMI count, mitochondrial gene ratio, ribosomal gene ratio, and erythrocyte gene ratio for the original expression matrix(a), the matrix after removing doublets(b), and the matrix filtered according to quality control criteria(c). d The dot plot of GO enrichment analysis of DEGs between the HC and STEMI groups. e The violon plots for the expression matrix after removing genes related to ribosomes, mitochondria, and erythrocytes. f The cell cycle scores for the expression matrix. Online Resource 4 The DEGs of subclusters 0, 1 and 6 of monocytes between STEMI and HC (top 10). Online Resource 5 a-c The top 5 GO enrichment terms of BP, CC and MF for the DEGs in subclusters 0 (a), 1 (b) and 6 (c) in monocytes. Online Resource 6 The heatmap of the GSVA scores in the 50 gene sets from the Hallmark database for T cells across samples in HC and STEMI groups. [file 41065_2025_609_MOESM1_ESM.zip › Additional file 1-6/Additional file 6.pdf]
